# Demystifying the Suprazygomatic Maxillary Nerve Block in Paediatric Cleft Palate Surgery

**DOI:** 10.1177/10556656241284514

**Published:** 2024-09-10

**Authors:** Matthew Fell, Lynn Fenner, Nefer Fallico

**Affiliations:** 1 Spires Cleft Lip and Palate Centre, 158997 Salisbury District Hospital, Salisbury, UK; 2 Department of Anaesthetics, 158997 Salisbury District Hospital, Salisbury, UK

**Keywords:** cleft palate, anatomy, palatoplasty

## Abstract

**Objective:**

To consider the clinical anatomy, safety and effectiveness of the suprazygomatic maxillary nerve block in cleft palate surgery.

**Design:**

Observational case series.

**Setting:**

Single cleft centre in the United Kingdom

**Participants:**

Patients born with a cleft palate (with or without a cleft lip) undergoing palatal surgery between the ages of 9 months and 18 years.

**Intervention:**

Introduction of suprazygomatic maxillary nerve (SZMN) block using ropivacaine 0.2% into clinical protocol in February 2023.

**Main Outcome Measures:**

Peri-procedure complications and post-operative opioid administration.

**Results:**

The clinical anatomy of the SZMN block is described in a stepwise and pictorial approach from superficial to deep structures. 43 patients underwent surgical interventions involving the palate (either intravelar veloplasty, Furlow palatoplasty or bilateral myomucosal buccinator flaps for palatal lengthening). 22 patients had a general anaesthetic and local anaesthetic infiltration and 21 had an additional SZMN block. There were no local or systemic complications associated with the SZMN block. There was no difference in the total dosing of post-operative (*P* = .79) opioids between the groups.

**Conclusions:**

We demonstrate the feasibility and safety of this procedure without the use of ultrasound guidance in a heterogenous group of paediatric patients undergoing palatal surgery. Regional anaesthesia should be considered as part of the multi-modal analgesic strategy, although it may be difficult to demonstrate a change in opioid use in clinical settings where enhanced recovery techniques are established, and opioid use is already low.

## Background

Perioperative pain in cleft palate surgery is inevitable due to the stimulation of the sensory nerve fibres following palatal mucosa incision and dissection. People born with a cleft palate undergo palatal surgery during childhood, with primary cleft palate repairs routinely offered between 9 and 13 months of age in the United Kingdom.^1^ The intra-oral location of the operation, in combination with the extremely young age of the majority of these patients, gives rise to potential complications of airway obstruction and respiratory distress.^2^ Opioid analgesia is routinely offered to reduce intra and postoperative pain, however it can exacerbate respiratory depression and infants are particularly susceptible to this due to immaturity of hepatic clearance mechanisms.^3^ Regional anaesthesia is appealing in palatal surgery because of a reduced risk of respiratory depression compared to opioid analgesia use. It is established that the multimodal combination of local, regional and general anaesthesia, if safe and feasible, is preferable in comparison to a single modality.^4^

Pre-incisional blockade of the maxillary nerve has been shown to produce effective intra- and post-operative analgesia for the soft and hard palate and has been in clinical use since the 1980s.^5^ Despite this, there has not yet been widespread adoption of the technique amongst cleft surgical teams and part of the reason for this may be the complexity of the anatomy of the structures involved.^6,7^

Maxillary nerve blocks have been described via both intraoral and extraoral approaches. The intra-oral approach is challenging due to the lack of depth recognition and a risk of passing the needle beyond the sphenoid bone.^8^ Extraoral approaches include the infrazygomatic and suprazygomatic techniques. The infrazygomatic approach is associated with a risk of injury to the orbit, maxillary artery and pharyngeal wall.^9^ The suprazygomatic maxillary nerve (SZMN) block is most commonly utilised in paediatric patients due to being technically easier to perform and safer, due to the reduced risk of iatrogenic injury to the orbit or maxillary artery.^2^

The SZMN block was introduced into the clinical protocol for palate surgery in a single cleft centre in the United Kingdom in February 2023. The aim of this service evaluation was threefold: first to describe the clinical anatomy of the maxillary nerve as it passes through the pterygopalatine fossa to increase familiarity for the injecting physician. Second, to assess the safety profile of the SZMN block by reporting any adverse events. Third, to observe the efficacy of the SZMN block in a non-research setting by assessing the intra-operative opioid use, time to first post-operative opioid administration and overall post-operative opioid administration.

## Clinical Anatomy of the Suprazygomatic Maxillary Nerve Block

**Figure 1.**
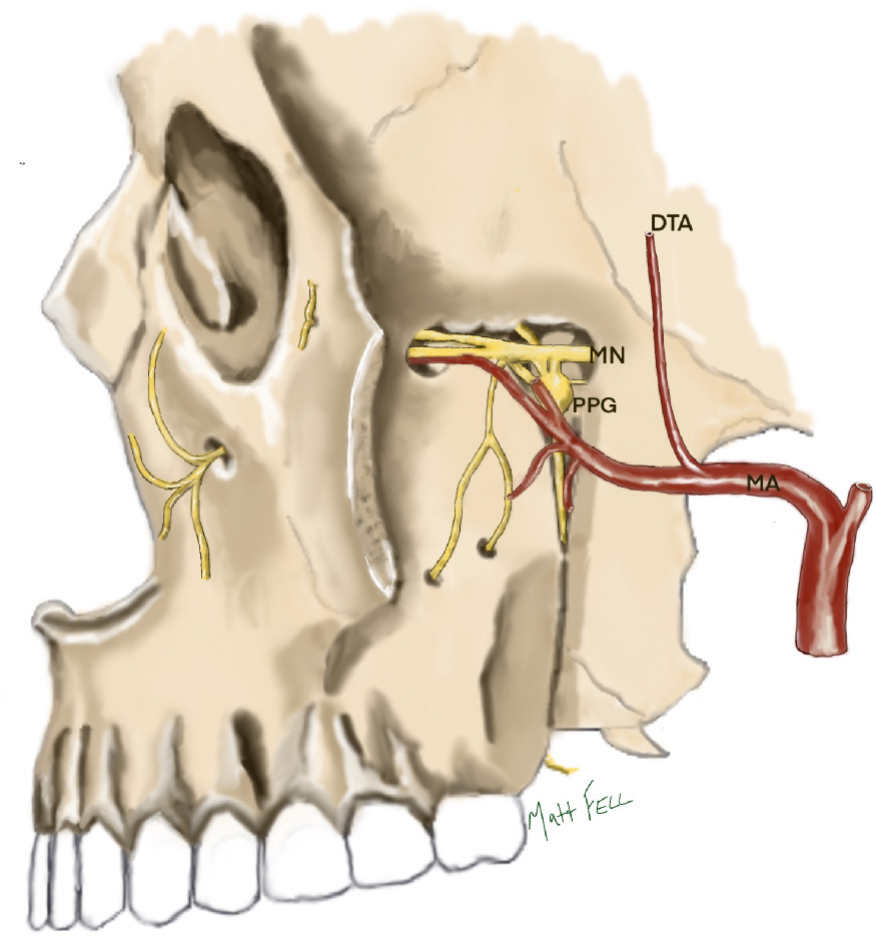
The contents of the pterygopalatine fossa. The maxillary nerve (MN) is situated in the superior aspect of the fossa, entering posteriorly through the foramen rotundum and exiting anteriorly through the inferior orbital fissure. The pterygopalatine ganglion (PPG) lies inferiorly to the maxillary nerve and is connected to it via short ganglionic nerve branches. The terminal branches of the maxillary artery (MA) enter the fossa from the medial aspect through the pterygomaxillary fissure. The main body of the maxillary artery lies ventrally and inferiorly to the maxillary nerve and pterygopalatine ganglion and the deep temporal artery (DTA) lies posteriorly.

The SZMN block involves the insertion of a needle through an avascular corridor in three anatomical planes; from the skin, through the infratemporal fossa to reach the pteryogopalatine fossa, which contains the maxillary nerve and pterygopalatine ganglion (See Figure 1).

**Figure 2.**
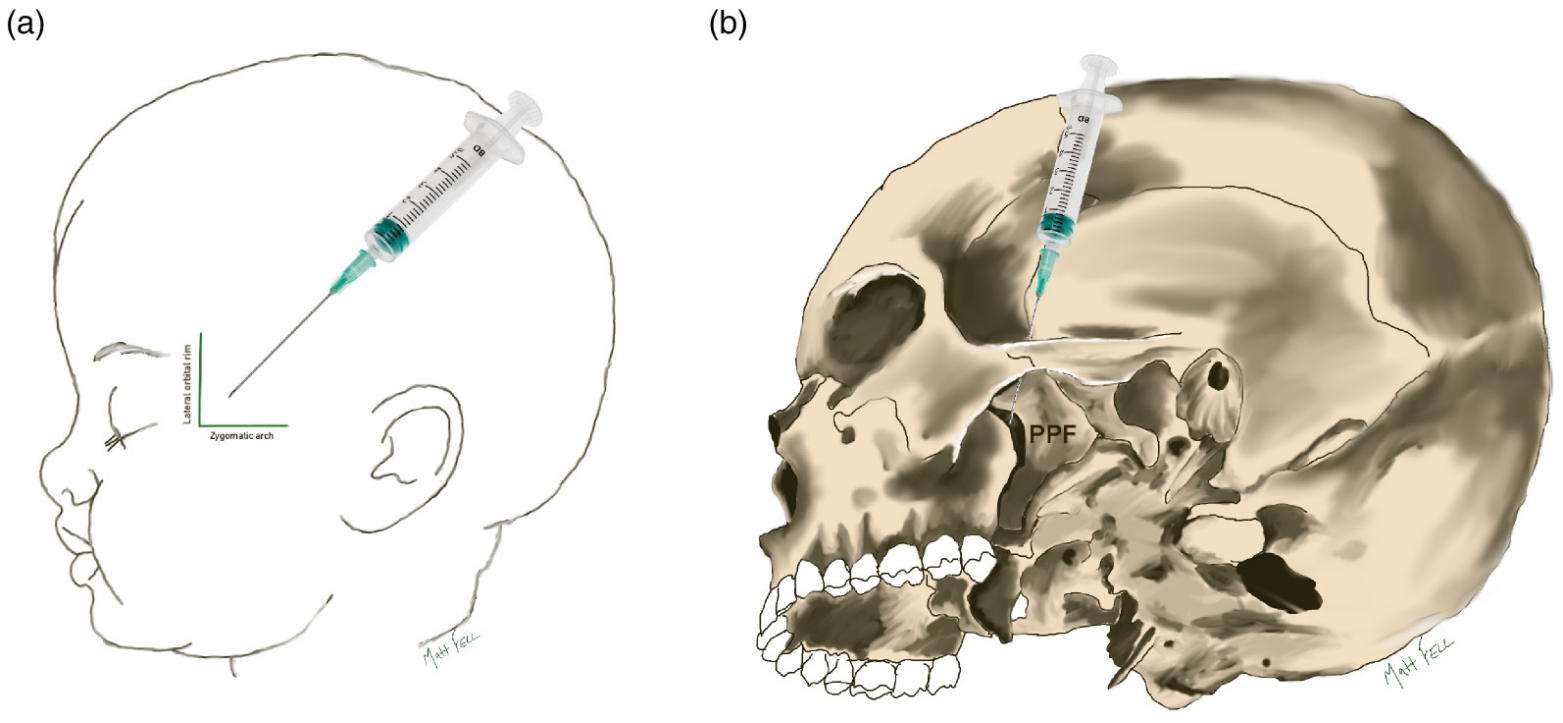
The orientation of the suprazygomatic nerve block in relation to the soft tissue (A) and bony skeleton (B). The needle in the soft tissue space behind the frontozygomatic angle, which is located at the intersection of the lateral orbital rim and zygomatic arch. The tip of the needle aims to reach the medial aspect of the superior half of the pterygopalatine fossa (PPF).

**Figure 3.**
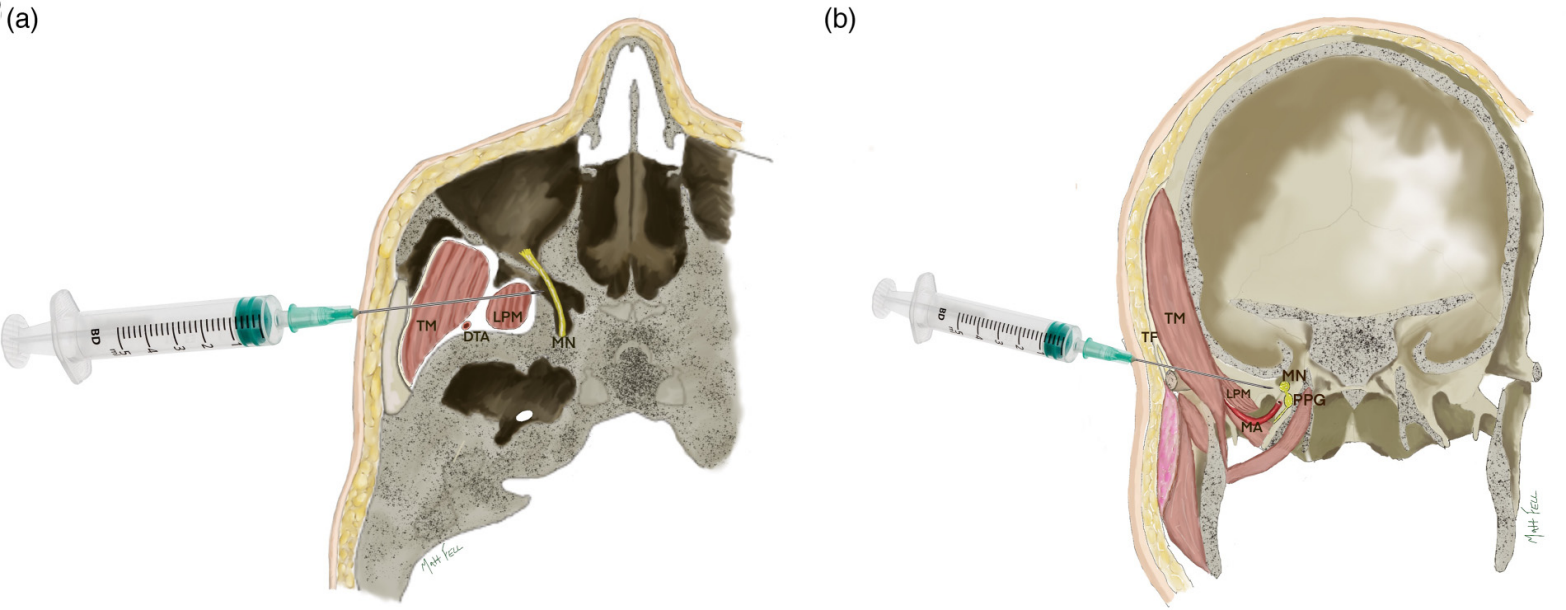
Axial (A) and coronal (B) slices to show the passage of the needle through the infratemporal fossa to reach the medial aspect of the pterygopalatine fossa containing the maxillary nerve (MN) and pterygopalatine ganglion (PPG). The needle passes through the temporal fascia (TF), the temporal muscle (TM) and the superior head of the lateral pterygoid muscle (LPM). The deep temporal artery is posterior (shown in 3A) and the main maxillary artery is inferior (shown in 3B) to the passage of the needle.

1. Skin: The surface landmark for the SZMN block is the frontozygomatic angle, which is positioned at the intersection of the lateral orbital rim and zygomatic arch (see Figure 2).^10^
2. Infratemporal fossa: The infratemporal fossa is the intermediate plane between the skin (as the needle passes beyond the zygomatic arch) and pterygopalatine fossa. It contains the muscles of mastication, branches of the maxillary artery and vein and the pterygoid venous plexus. The temporalis muscle occupies most of the space in the lateral aspect of the infratemporal fossa and is covered by the temporalis fascia, which explains the initial resistance encountered by the needle. Medial to the temporalis muscle is the lateral pterygoid muscle. The needle in the SZMN block passes through the superior head of the lateral pterygoid muscle, as it is advanced towards the superior aspect of the pterygopalatine fossa where the maxillary nerve lies (see Figure 3). Of note, the maxillary artery is positioned more inferiorly as it enters the pterygopalatine fossa, by passing between the superior and inferior heads of the lateral pterygoid muscle. The deep temporal artery is a main branch and is positioned posteriorly to the pterygopalatine fossa.
3. Pterygoplalatine fossa: The pterygopalatine fossa is a small space in the shape of an inverted pyramid that lies deep within the face at the base of the skull bilaterally and is an important anatomical crossroads, containing the maxillary nerve, the pterygopalatine ganglion and terminal branches of the maxillary artery. The space is bordered by the body of the maxilla anteriorly, the lateral pterygoid plate posteriorly, the greater wing of sphenoid superiorly and the perpendicular lamina of the palatine bone medially.^10^ The maxillary nerve and its branches lie in the superior half of the fossa, the pterygopalatine ganglion is suspended beneath it and the vascular branches of the maxillary artery are located inferiorly and posteriorly.

The maxillary nerve is the target of the SZMN block. It is the second division of the trigeminal nerve, it is a purely sensory nerve and supplies sensation to the midface including the palate.^11^ Specifically, sensation to the hard and soft palate is provided via three branches of the maxillary nerve which are the greater palatine nerve (hard palate), the lesser palatine nerve (soft palate) and the nasopalatine nerve (anterior hard palate from canine to canine).^2,12^ The pterygopalatine ganglion is a large parasympathetic structure which involves the transit of autonomic, motor and sensory nerves and is located just inferiorly to the maxillary nerve, connecting to it with short ganglionic branches.^13^ The greater and lesser palatine and nasopalatine nerves all pass through the pterygopalatine ganglion without synapsing to reach the maxillary nerve.^14^ The SZMN block technique aims to infiltrate a local anaesthetic agent in the proximity of the maxillary nerve and pterygopalatine ganglion, to prevent the conduction of nociceptive impulses through the sensory nerves.^4^

## Method

### Participants

Included in this study were consecutive paediatric patients aged between 9 months and 18 years, born with a cleft palate who underwent palatal surgery at the Salisbury site of the Spires Cleft Centre UK between June 2022 and November 2023. The control group (general anaesthesia and local anaesthetic infiltration at operative site) consisted of consecutive palatoplasties performed between June 2022 and January 2023, prior to the introduction of the SZMN block in clinical practice. The block group consisted of patients undergoing palatoplasties performed between February and November 2023 (SZMN block in addition to general anaesthetic and local anaesthetic infiltration). All cleft palate phenotypes were included (overt cleft palate and submucous cleft palate) and there were no exclusions for patients born with syndromes or additional congenital anomalies. Palatal surgery included primary cleft palate reconstruction (intravelar veloplasty for overt cleft palate reconstruction or Furlow palatoplasty for submucous cleft palate reconstruction) or secondary palatal lengthening for speech (interpositional buccinator myomucosal flaps).

### Bilateral Suprazygomatic Nerve Block Technique

The SZMN block was administered bilaterally by the operating surgeon (NF) using the anatomical landmark technique in the anaesthetised patient at the start of the case before sterile draping. 4 mls of 0.2% Ropivacaine is drawn up into a 5 ml syringe (2 mls to be used on each side) mounted with a 21 gauge, 40 mm needle. The patient is positioned supine with head neutral.

The entry point for the needle is approximately 5 mm posterior to the frontozygomatic angle in the soft tissue space and the needle is orientated anteriorly and inferiorly, aiming towards the contralateral tragus (See Figure 2).

Once through the skin and subcutaneous fat, the operator feels resistance from the temporalis fascia and the ‘give’ when the needle passes through the temporalis and lateral pterygoid muscles.

Advancing the 40 mm needle to its hilt places the tip just lateral to the pterygopalatine fossa and its contents. Negative pressure is applied to ensure against inadvertent cannulation of the maxillary artery or one of its branches. 2 mls 0.2% Ropivicaine is infiltrated slowly, the needle withdrawn, and surface pressure applied with a gauze. A transparent dressing is applied to facilitate observation for skin changes or swelling and any systemic changes observed for via the standard monitoring.

### Peri-operative Protocol

Patients undergoing palatal surgery were operated by the senior author (NF). Monitoring, including non-invasive blood pressure, electrocardiograph, pulse oximeter and end-tidal CO2, was used during the perioperative period. General anaesthesia, administered by one of five designated consultant cleft anaesthetists, was induced using gas induction for younger children and IV induction was preferred for older children. Children underwent endotracheal intubation with a cuffed tube and mechanical ventilation was applied. Anaesthesia was maintained with inhaled sevoflurane by four anaesthetists and total intravenous anaesthesia (TIVA) by one anaesthetist. Antibiotics (co-amoxiclav as a first line in the absence of allergies), tranexamic acid (10 mg/kg) and dexamethasone (0.25 mg/kg) were given on induction. Background intra-operative analgesia was provided by intravenous paracetamol and rectal diclofenac (for children more than 6 months of age and more than 8 kg in weight). There was variation in opioid use with the three main intraoperative regimens being: fentanyl 1-2 mcg/kg, followed by morphine 0.1 mg.kg; fentanyl 1-3 mcg/kg, or remifentanil (if TIVA used) followed by fentanyl 1-3 mcg/kg.

Pre-incisional local anaesthetic solution was infiltrated into the operating site within the palatal mucosa using 0.25% Bupivacaine with adrenaline, appropriately dosed by the child's weight. Surgical techniques for the three operative procedures used in this study followed previous descriptions.^15–17^ Post-operatively, patients were admitted to the paediatric ward for monitoring overnight before being discharged home the following day. Regular analgesia was provided with paracetamol, ibuprofen and benzylamine hydrochloride spray. Breakthrough pain was treated with oral morphine.

### Outcomes

The primary outcome was the observation of complications relating to the SZMN block. Patients underwent observation for complications during their inpatient admission and were reviewed face-to-face at 1 week by the cleft clinical nurse specialist and at 6 weeks post-operatively by the cleft surgeon (NF) in the cleft clinic. Data was recorded prospectively by the single operating surgeon (NF) who administered the bilateral SZMN block.

The secondary outcome was the administration of post-operative opioid analgesics and data was collected retrospectively by chart review. We recorded whether post-operative opioids were administered, if so, time to first dose and total quantity of opioid per Kg of child weight.

### Data Analysis

Categorical data was reported with frequencies and percentages. Continuous data was assessed for fit to a normal distribution with a histogram and was reported as mean (standard deviation) if normally distributed and median (interquartile range) if non-normally distributed. An unpaired t-test was used to assess the difference for continuous data between the control and intervention cohorts.^18^ P values were reported and interpreted as continuous measures of the strength of evidence against the null hypothesis.^19^ Due to inequalities in age of participants between the control and block groups, a supplementary post-hoc analysis was carried out to compare post-operative opioid requirements in patients undergoing primary palatoplasty for overt cleft palate in infancy versus submucous cleft palate reconstruction and secondary speech surgery in childhood.

### Ethical Considerations

This observational case-series was identified as a service evaluation with the Clinical Audit and Transformation Team at Salisbury District Hospital, UK and ethical approval was not required.^20^

## Results

43 patients born with a cleft palate were included in this study; 22 in the control group and 21 in the block group. Patient ages at operation ranged from 10 months to 17 years. There were 17 males and 27 females. Cleft palate subtypes included cleft palate only (19), cleft lip and palate (12) and submucous cleft palate (11). Operative interventions included intravelar veloplasty for overt cleft palate (17), Furlow palatoplasty for submucous cleft palate (11), and bilateral myomucosal buccinator flaps for speech (15). Gas induction was used for 32 patients and IV induction in 11 patients. Inhaled sevoflurane was used for maintenance in 39 patients and TIVA in 4 patients. Intraoperative opioid analgesia was achieved with intravenous fentanyl in 30 cases and morphine in 13 cases. All patients were successfully discharged home on post-operative day 1. Demographic details for included patients are detailed in Table 1.

**Table 1.** Demographic Details of Consecutive Patients Included in This Study.

Variable	GA and local anaesthetic (Total 22)	Additional SZMN block (Total 21)
Age	Median 83 months (IQR: 91)	Median 12 months (IQR: 61)
Sex	Males 10 (46%)	Males 7 (33%)
	Females 12 (54%)	Females 13 (66%)
Cleft type	Submucous (7)	Submucous (4)
	Veau 1 CPO (5)	Veau 1 CPO (5)
	Veau 2 CPO (4)	Veau 2 CPO (5)
	UCLP (3)	UCLP (6)
	BCLP (2)	BCLP (1)
Operation	Furlow (7)	Furlow (4)
	IVVP (5)	IVVP (12)
	Buccinators (10)	Buccinators (5)

The SZMN block was successfully administered bilaterally in 21 patients undergoing consecutive palatal surgery. There were no local or systemic complications associated with the SZMN block in any of the 21 patients during the inpatient admission or within the 6-week follow-up period.

In the control group 11/22 (50%) did not require post operative breakthrough analgesia with morphine and of the 11 that did, the mean time to first opioid was 5.45 h (SD 3.78). In the block group 9/21 (43%) did not require post operative breakthrough analgesia with morphine and of the 12 that did, the mean time to first opioid was 5.3 h (SD 3.98). There was no evidence for a difference in time to first opioid between the control group and block group (T = 0.09; *P* = .93). The mean total post-operative dose of morphine was 0.16 mg/kg (SD 0.23) in the control group was comparable to 0.14 mg/kg (SD 0.16) in the block group (T = 0.27; *P* = .79).

In the supplementary post-hoc analysis, 18 were in the infant age group (undergoing primary palatoplasty for an overt cleft palate) with a median age of 11 months and 25 were in the childhood age group (undergoing reconstruction for submucous cleft palate or secondary speech surgery) with a median age of 7 years and 7 months. In the infant group, 6/18 (33%) did not require post-operative breakthrough analgesia with morphine and of the 12 that did, the mean time to first opioid was 5.77 h (SD 3.80). In the childhood group, 15/25 (60%) did not require post operative breakthrough analgesia with morphine and of the 10 that did the mean time to first opioid was 4.95 h (SD 3.90). There was no evidence for a difference in time to first opioid between the infant and childhood groups (T = 0.49; *P* = .32). The mean total post-operative dose of morphine in the infant group was 0.23 mg/kg (SD 0.25), compared to 0.10 mg/kg (SD 0.14) in the childhood group, but variation in the data resulted in weak evidence for any difference between the groups (T = 1.84; *P* = .08).

## Discussion

### Key Findings

The pterygopalatine fossa is an important anatomical crossroads and offers the potential for regional anaesthetic blockade in cleft palate surgery due to it containing the maxillary nerve and pterygopalatine ganglion, which transmit sensory nerve fibres supplying the hard and soft palate. We describe the layered anatomy of the SZMN block, from the skin down to the pterygopalatine fossa, and its introduction into the surgical pathway for paediatric patients undergoing palatal surgery in a specialised UK cleft unit. The change in practice was successful in terms of the block being easy to administer and safe, with an absence of any observed complications. There was no demonstrable difference in the use of post-operative opioid analgesics in our clinical setting after the introduction of the SZMN block.

### Comparison to Other Studies

The anatomy of the pterygopalatine fossa and its contents are complex because they involve numerous branching neurovascular structures, which are deeply located near the base of the skull, yet they have been well described in cadaveric and radiological studies.^13,21–24^ Series of CT scans demonstrate anatomical consistency and consensus across paediatric populations in terms of the depth of the pterygopalatine fossa from the frontozygomatic angle and the required orientation for the SZMN block needle.^4,25^ Whilst we have used these robust findings to inform the technique reported in this study, the interpretation of paediatric CT images requires specialist interpretation and may not be user-friendly for cleft clinicians. We have supplemented the literature in this study with anatomical diagrams to demystify the anatomy in a stepwise manner, which we hope will be useful for physicians who feel less familiar in this territory.

Potential complications of the SZMN block have been cited as oedema, nausea, vomiting, haematoma, sedation, pupil alteration, ocular injury and systemic toxicity, however the safety of the technique has been widely emphasised.^2,26^ Smith et al. (2022)^7^ reported a large series of 411 adult patients having the SZMN block and only identified a minor complication of post-injection ooze in one patient. Previous authors have described manoeuvres to try and reduce the complication risk such as directing the needle perpendicular over the zygomatic arch to hit the greater wing of sphenoid before re-orientating under the wing towards the pterygopalatine fossa.^4^ We feel this two-stage needling approach is unnecessary because the anatomy has been shown to be consistent and a direct approach from the skin down to the PPF using anatomical landmarks is less traumatic. Furthermore, anatomical injection studies have shown the orientation of the needle aiming for the contralateral tragus to be advantageous for positioning the tip pf the needle closest to the entrance of the pterygopalatine fossa.^27^

The use of ultrasound guidance has been described in the literature, but this adds complexity in terms of additional equipment and time in the operating theatre and furthermore, a proportion of ultrasounds have been shown to be futile, due to poor obtained images or a failure to observe the spread of the local anaesthetic.^28^ We perform the block as originally described, using anatomical landmarks, which we and others have shown to be safe and which minimises delays to the operative procedure.^29^

The efficacy of the SZMN block has been evaluated in seven comparative studies to date (see Table 2) and consensus from systematic reviews in this field is that the block effectively reduces pain and opioid use.^26,30^ The earliest trial in France by Chiono et al. (2014)^9^ compared the SZMN block to GA only and reported a reduction in post-operative morphine use, but no difference in pain scores or time to feed. Elyazed and Mostafa (2018)^2^ in an Egyptian study compared the SZMN to GA and local anaesthetic infiltration and reported lower intra-operative fentanyl, lower pain scores, shorter time to feed and lowest need for rescue analgesia in the block group. In comparison, we have not been able to demonstrate a difference in post-operative opioid administration in our clinical setting following the introduction of the SZMN block. Our findings are similar to Binet et al. (2015)^31^ who published a historical cohort comparison of GA alone versus SZMN block for children undergoing primary palatoplasty and did not find a statistically significant decrease in the peri-operative use of morphine.

**Table 2.** Comparative Studies Investigating Suprazygomatic Maxillary Nerve Block in the Cleft Palate Population.

Author	Country	Population	Study design	Patient no.	Intervention	Control	Outcome
Chiono 2014	France	Primary palatoplasty mean age 18-22 months (range not provided)	Randomized control trial	60	Bilateral SZMN block with ropivacaine 0.2%	GA only with placebo injection of saline	Post-operative morphine: SZMN block reduced by 50%. Pain score: no difference. Time to feed: no difference. 2 complications of bleeding in SZMN block group
Mostafa 2018	Egypt	Primary palatoplasty age 1-10 years	Randomized control trial	60	Bilateral SZMN block with Levobupivacaine 0.2%	Bilateral SZMN block with bupivacaine 0.2%	No difference in any outcome including pain scores, time to first opioid dose and total dose of opioids
Elyazed and Mostafa 2018	Egypt	Primary palatoplasty age 3 months - 2 years	Randomized control trial	90	Bilateral SZMN block with bupivacaine 0.25%	GA only and GA with local anaesthetic infiltration with bupivacaine 0.25%	Intra-op fentanyl: lowest for SZMN block. Pain score: lowest for SZMN block. Need for rescue opioids: lowest for SZMN block. Time to feed: shortest for SZMN nerve block. No significant complications noted
Parameswaran et al. 2018	India	Primary palatoplasty age 10 months to 14 years	Randomized control trial	97	Transoral pterygopalatine ganglion block with 0.75% ropivacaine	GA only	Pain score: no difference. Post-op pain free duration: longer for SPG block. Intra-operative heart rate: no difference
Echaniz et al., 2019	India	Primary palatoplasty, median age 3-5 years (no range provided)	Randomized control trial	34	Bilateral SZMN block with bupivacaine 0.25% and adrenaline 1: 200 000	Palatine nerve block with 0.25% bupivacaine and adrenaline 1: 200 000	Percentage of patients requiring post-op opioids: no difference (234% for SZM versus 17% for palatine). Intra-op fentanyl: reduced in SZMN
Mostafa 2020	Egypt	Primary palatoplasty age 1-5 years	Randomized control trial	80	Bilateral SZMN block with bupivacaine 0.125% and dexmedetomidine	Bilateral SZMN block with bupivacaine 0.125% alone	Pain score: lower in dexmedetomidine group. Blood pressure and heart rate: lower in dexmedetomidine group. Sedation score during first 30 min: higher in dexmedetomidine group. Patient satisfaction scores: higher in dexmedetomidine group. No serious adverse effects
Barbero 2021	India	Primary palatoplasty: median age 30-33 months (no range)	Randomized control trial	54	Bilateral SZMN block with bupivacaine 0.25% and clonidine mixture	Bilateral SZMN block with bupivacaine 0.25% alone	Use of intra-op fentanyl lower in clonidine group 10% versus 40%. Extra opioids: lower in clonidine 17% vs 48%. No difference in rescue propofol, nalbuphine intro or post-op, emergence agitation and length of stay

### Interpretation

The safety of the SZMN block aligns with our knowledge of the anatomy of this region. The risk of cannulating foramina and entering either the orbital or cranial cavities is minimised by the orientation and placement of the needle in the suprazygomatic approach.^4^ There is a realistic risk of penetrating the maxillary artery or the pterygoid venous plexus, yet both these structures lie ventral and inferior to the maxillary nerve and with negative pressure applied prior to infiltrating the anaesthetic, this risk is minimised.

Our findings of equivalent opioid use following the introduction of the SZMN block is perhaps not surprising in this clinical setting. First, the introduction of the regional block was an additional analgesic modality within an established multi-modal strategy. Opioid use was already low prior to the introduction of the SZMN block, probably due to use of modern anaesthetic and surgical techniques, long-acting local anaesthetic infiltration at the operative site and enhanced recovery strategies. Any further reductions would likely have been subtle. Previous comparative studies in the literature have been conflicted in their reported findings regarding the influence of long-acting local anaesthetic infiltration at the operative site to overshadow the impact of the SZMN block (See Table 2). Elyazed and Mostafa (2018)^2^ reported lower post-operative opioid requirements in patients with SZMN compared to local 0.25% bupivacaine infiltration, whereas Echaniz et al. (2019)^11^ reported no difference in the proportion of patients requiring post-operative opioids following the SZMN block versus local bupivacaine 0.25% and adrenaline infiltration. Second, this and other related studies highlight the challenges involved with monitoring post-operative pain in the paediatric population. Multiple options are available including opioid use, pain scores and post-operative feeding, yet there is an absence of a robust method that could be considered gold standard. Opioid administration is the most commonly used outcome measure in the literature, yet the administration of opioids is a subjective clinical decision and is open to individual variation which is difficult to standardise.

We believe the SZMN block offers an elegant opportunity to provide regional anaesthesia of the palatal nerves in addition to systemic and local modalities. The convincing safety profile of the block makes this an attractive option as part of the wider efforts to enhance recovery after surgery, to improve the peri-operative pathway and improve post-operative outcomes.

### Strengths, Limitations and Further Work

This study was carried out in a specialised clinical cleft unit and therefore represents an experience that can be used by other clinical teams in similar environments. The heterogeneity of the patients, procedures and anaesthetists is more realistic than the controlled conditions within a research environment, yet this presents challenges for data interpretation. In particular, the ages of the children were different between the two comparative groups in this study and the variety of anaesthetic and analgesic agents used by the five paediatric cleft anaesthetics demonstrates the variability within our cohort. Our supplementary post-hoc analysis of infant versus childhood age groups aimed to help understand the risk of the age inequality and whilst there was weak evidence of a statistical difference in opioid requirement between the age groups, there was a trend for the childhood group to be less likely to require breakthrough opioids and for those that did require it, for the total dose of opioid to be less. This may have masked the impact of the introduction of the SZMN block and its ability to reduce post-operative opioid requirements. Anaesthetic agent variations reflected individual preferences of the anaesthetists and could have confounded the post-operative outcomes. The small sample size in this study reduced the power of the statistical analysis. We were limited in the range of outcomes we could measure due to inconsistencies in the collection of additional outcomes in our centre that have previously been published in the literature, such as pain scores and time to first feed. Further work is required to understand the impact of the SZMN block, both in our clinical setting and wider clinical settings, with a consensus on objective outcome measures needed.

## Conclusion

To summarize our findings according to the three initial objectives: First we have described the clinical anatomy of the maxillary nerve as it passes through the pterygopalatine fossa in a stepwise pictorial way to increase familiarity for the injecting physician. Second, we have found the SZMN block to be safe since its introduction into the cleft palate surgical pathway with no observed complications. Third, despite the inability to demonstrate a reduction in post-operative opioids following the introduction of the SZMN block in our cohort, we believe its use should be considered as part of the multi-model analgesia strategy in paediatric cleft palate surgery.

## References

[1] Fell M Davies A Davies A , et al. Current surgical practice for children born with a cleft lip and/or palate in the United Kingdom. Cleft Palate Craniofac J. 2023;60(6):679‐688. 35199604 10.1177/10556656221078151

[2] Abu Elyazed MM Mostafa SF . Bilateral suprazygomatic maxillary nerve block versus palatal block for cleft palate repair in children: A randomized controlled trial. Egypt J Anaesth. 2018;34(3):83‐88.

[3] Rossell-Perry P Romero-Narvaez C Rojas-Sandoval R Gomez-Henao P Delgado-Jimenez MP Marca-Ticona R . Is the use of opioids safe after primary cleft palate repair? A systematic review. Plast Reconstr Surg Glob Open. 2021;9(1):e3355. 10.1097/GOX.0000000000003355 PMC7858197 33564585

[4] Captier G Dadure C Leboucq N Sagintaah M Canaud N . Anatomic study using three-dimensional computed tomographic scan measurement for truncal maxillary nerve blocks via the suprazygomatic route in infants. J Craniofac Surg. 2009;20(1):224‐228. 19165032 10.1097/SCS.0b013e318191d067

[5] Cometa MA Zasimovich Y Smith CR . Sphenopalatine ganglion block: Do not give up on it just yet!. Br J Anaesth. 2021;126(6):e198‐e200. 10.1016/j.bja.2021.02.020 33795136

[6] Tanner JA Jethwa B Jackson J , et al. A three-dimensional print model of the pterygopalatine Fossa significantly enhances the learning experience. Anat Sci Educ. 2020;13(5):568‐580. 31904166 10.1002/ase.1942

[7] Smith CR Dickinson KJ Carrazana G , et al. Ultrasound-Guided suprazygomatic nerve blocks to the pterygopalatine Fossa: A safe procedure. Pain Med. 2022;23(8):1366‐1375. 35043949 10.1093/pm/pnac007 PMC9608014

[8] Parameswaran A Ganeshmurthy MV Ashok Y Ramanathan M Markus AF Sailer HF . Does sphenopalatine ganglion block improve pain control and intraoperative hemodynamics in children undergoing palatoplasty? A randomized controlled trial. J Oral Maxillofac Surg. 2018;76(9):1873‐1881. 29684306 10.1016/j.joms.2018.03.037

[9] Chiono J Raux O Bringuier S , et al. Bilateral suprazygomatic maxillary nerve block for cleft palate repair in children: A prospective, randomized, double-blind study versus placebo. Anesthesiology. 2014;120(6):1362‐1369. 24525630 10.1097/ALN.0000000000000171

[10] Derinkuyu BE Boyunaga O Oztunali C Alimli AG Ucar M . Pterygopalatine Fossa: Not a mystery!. Can Assoc Radiol J. 2017;68(2):122‐130. 27932266 10.1016/j.carj.2016.08.001

[11] Echaniz G De Miguel M Merritt G , et al. Bilateral suprazygomatic maxillary nerve blocks vs. infraorbital and palatine nerve blocks in cleft lip and palate repair: A double-blind, randomised study. Eur J Anaesthesiol. 2019;36(1):40‐47. 30308523 10.1097/EJA.0000000000000900

[12] Nores GDG Cuzzone DA Hush SE , et al. The impact of bilateral suprazygomatic maxillary nerve blocks on postoperative pain control in patients undergoing orthognathic surgery. Face. 2020;1(1):58‐65.

[13] Rusu MC Pop F Curcă GC Podoleanu L Voinea LM . The pterygopalatine ganglion in humans: A morphological study. Ann Anat. 2009;191(2):196‐202. 19124232 10.1016/j.aanat.2008.09.008

[14] Iwanaga J Wilson C Simonds E , et al. Clinical anatomy of blockade of the pterygopalatine ganglion: Literature review and pictorial tour using cadaveric images. Kurume Med J. 2018;65(1):1‐5. 30158355 10.2739/kurumemedj.MS651001

[15] Sommerlad BC . A technique for cleft palate repair. Plast Reconstr Surg. 2003;112(6):1542‐1548. 14578783 10.1097/01.PRS.0000085599.84458.D2

[16] Furlow LT Jr . Cleft palate repair by double opposing Z-plasty. Plast Reconstr Surg. 1986;78(6):724‐738. 3786527 10.1097/00006534-198678060-00002

[17] Hill C Hayden C Riaz M Leonard AG . Buccinator sandwich pushback: A new technique for treatment of secondary velopharyngeal incompetence. Cleft Palate Craniofac J. 2004;41(3):230‐237. 15151445 10.1597/02-146.1

[18] Parab S Bhalerao S . Choosing statistical test. Int J Ayurveda Res. 2010;1(3):187‐191. 21170214 10.4103/0974-7788.72494 PMC2996580

[19] Greenland S Senn SJ Rothman KJ , et al. Statistical tests, P values, confidence intervals, and power: A guide to misinterpretations. Eur J Epidemiol. 2016;31(4):337‐350. 27209009 10.1007/s10654-016-0149-3 PMC4877414

[20] NHS Health Research Authority. What approvals and decisions do I need? https://www.hra.nhs.uk/approvals-amendments/what-approvals-do-i-need/. Accessed 28th May 2024.

[21] Rusu MC Pop F . The anatomy of the sympathetic pathway through the pterygopalatine fossa in humans. Ann Anat. 2010;192(1):17‐22. 19939656 10.1016/j.aanat.2009.10.003

[22] Rusu MC Didilescu AC Jianu AM Păduraru D . 3D CBCT anatomy of the pterygopalatine fossa. Surg Radiol Anat. 2013;35(2):143‐159. 22918475 10.1007/s00276-012-1009-9

[23] Prigge L van Schoor AN Bosman MC Bosenberg AT . Clinical anatomy of the maxillary nerve block in pediatric patients. Paediatr Anaesth. 2014;24(11):1120‐1126. 25040918 10.1111/pan.12480

[24] Gibelli D Cellina M Gibelli S , et al. Anatomy of the pterygopalatine fossa: An innovative metrical assessment based on 3D segmentation on head CT-scan. Surg Radiol Anat. 2019;41(5):523‐528. 30542926 10.1007/s00276-018-2153-7

[25] Marston AP Merritt G Morris JM Cofer SA . Impact of age on the anatomy of the pediatric pterygopalatine fossa and its relationship to the suprazygomatic maxillary nerve block. Int J Pediatr Otorhinolaryngol. 2018;105:85‐89. 29447826 10.1016/j.ijporl.2017.12.012

[26] Morzycki A Nickel K Newton D Ng MC Guilfoyle R . In search of the optimal pain management strategy for children undergoing cleft lip and palate repair: A systematic review and meta-analysis. J Plast Reconstr Aesthet Surg. 2022;75(11):4221‐4232. 36171173 10.1016/j.bjps.2022.06.104

[27] Peters JJ Jacobs K Munill M , et al. The maxillary nerve block in cleft palate care: A review of the literature and expert’s opinion on the preferred technique of administration. J Craniofac Surg. 2024;35(5):1356‐1363. 38861198 10.1097/SCS.0000000000010343 PMC11198960

[28] Sola C Raux O Savath L Macq C Capdevila X Dadure C . Ultrasound guidance characteristics and efficiency of suprazygomatic maxillary nerve blocks in infants: A descriptive prospective study. Paediatr Anaesth. 2012;22(9):841‐846. 22587691 10.1111/j.1460-9592.2012.03861.x

[29] Mireault D Cawthorn TR Todd AR Spencer AO . Suprazygomatic maxillary nerve block: An ultrasound and cadaveric study to identify correct sonoanatomical landmarks. J Anesth. 2021;35(1):150‐153. 33230676 10.1007/s00540-020-02877-6

[30] Pfaff MJ Nolan IT Musavi L , et al. Perioperative pain management in cleft lip and palate surgery: A systematic review and meta-analysis of randomized controlled studies. Plast Reconstr Surg. 2022;150(1):145e‐156e. 10.1097/PRS.0000000000009231 35579433

[31] Binet A Thinnes J Amory C , et al. Role of bilateral suprazygomatic maxillary nerve block in primary surgery for soft palate cleft and soft-hard palate cleft. Ped Anesth Crit Care J. 2015;3(2):111‐117.

